# Detailed statistical analysis plan for the SafeBoosC III trial: a multinational randomised clinical trial assessing treatment guided by cerebral oxygenation monitoring versus treatment as usual in extremely preterm infants

**DOI:** 10.1186/s13063-019-3756-y

**Published:** 2019-12-19

**Authors:** Mathias Lühr Hansen, Adelina Pellicer, Christian Gluud, Eugene Dempsey, Jonathan Mintzer, Simon Hyttel-Sorensen, Anne Marie Heuchan, Cornelia Hagmann, Gabriel Dimitriou, Gerhard Pichler, Gunnar Naulaers, Guoqiang Cheng, Ana Vilan, Jakub Tkaczyk, Karen B. Kreutzer, Monica Fumagalli, Olivier Claris, Siv Fredly, Tomasz Szczapa, Theis Lange, Janus Christian Jakobsen, Gorm Greisen

**Affiliations:** 1 Department of Neonatology, Rigshospitalet, Copenhagen University Hospital, Blegdamsvej 9, 2100 Copenhagen, Denmark; 2 Department of Neonatology, La Paz University Hospital, Paseo De La Castellana 261, 28046 Madrid, Spain; 3 Copenhagen Trial Unit, Centre for Clinical Intervention Research, Rigshospitalet, Copenhagen University Hospital, Blegdamsvej 9, 2100 Copenhagen, Denmark; 4 Infant Centre and Department of Paediatrics and Child Health, University College Cork, College Road, Cork, Ireland; 5 Division of Neonatal-Perinatal Medicine, Mountainside Medical Center, Montclair, NJ USA; 6 Department of Neonatology, Royal Hospital for Children, 1345 Govan Rd, Glasgow, G51 4TF UK; 7 Department of Neonatology, Children’s University Hospital of Zürich, Steinweisstrasse 75, 8037 Zurich, Switzerland; 8 NICU, Department of Pediatrics, University General Hospital of Patras, 265 04 Patras, Greece; 9 Department of Pediatrics, Medical University of Graz, Auenbruggerplatz 30, Graz, Austria; 10 Department of Neonatology, University Hospital Leuven, Herestraat 49, Leuven, Belgium; 11 Department of Neonatology, Children’s Hospital of Fudan University, 399 Wanyuan Rd, Minhang Qu, Shanghai Shi, China; 12 Department of Neonatology, Centrohospitalar Universitário de São João, Alameda Prof. Hernâni Monteiro, 4200-319 Porto, Portugal; 13 Department of Neonatology, University Hospital Motol, V Uvalu 84, 150 06 Prague 5, Czech Republic; 14 Department of Neonatology, University Children’s Hospital Tuebingen, Hoppe-Seyler-Straße 1, 72076 Tuebingen, Germany; 15 Fondazione IRCCS Cà Granda Ospedale Maggiore Policlinico Milan, Via della Commenda 12, 20122 Milan, Italy; 16 Department of Clinical Sciences and Community Health, University of Milan, Milan, Italy; 17 Department of Neonatology, Hospices Civil De Lyon, 3 Quai des Célestins, 69002 Lyon, France; 18 Department of Neonatology, Oslo University Hospital, Kirkeveien, 166 0450 Oslo, Norway; 19 Department of Neonatology, Poznan University of Medical Sciences, Polna 33, 60-535 Poznań, Poland; 20 Section of Biostatistics, Department of Publich Health, Copenhagen University, Øster Farimagsgade 5, Copenhagen K, Denmark; 21 Center for Statistical Science, Peking University, Beijing, China; 22 Department of Cardiology, Holbæk Hospital, Smedelundsgade 60, 4300 Holbæk, Denmark; 23 Department of Regional Health Research, The Faculty of Health Sciences, University of Southern Denmark, Odense, Denmark

**Keywords:** Randomised clinical trial, Extremely preterm, Near-infrared spectroscopy, Cerebral oximetry, Statistical analysis plan

## Abstract

**Background:**

Infants born extremely preterm are at high risk of dying or suffering from severe brain injuries. Treatment guided by monitoring of cerebral oxygenation may reduce the risk of death and neurologic complications. The SafeBoosC III trial evaluates the effects of treatment guided by cerebral oxygenation monitoring versus treatment as usual. This article describes the detailed statistical analysis plan for the main publication, with the aim to prevent outcome reporting bias and data-driven analyses.

**Methods/design:**

The SafeBoosC III trial is an investigator-initiated, randomised, multinational, pragmatic phase III trial with a parallel group structure, designed to investigate the benefits and harms of treatment based on cerebral near-infrared spectroscopy monitoring compared with treatment as usual. Randomisation will be 1:1 stratified for neonatal intensive care unit and gestational age (lower gestational age (< 26 weeks) compared to higher gestational age (≥ 26 weeks)). The primary outcome is a composite of death or severe brain injury at 36 weeks postmenstrual age. Primary analysis will be made on the intention-to-treat population for all outcomes, using mixed-model logistic regression adjusting for stratification variables. In the primary analysis, the twin intra-class correlation coefficient will not be considered. However, we will perform sensitivity analyses to address this. Our simulation study suggests that the inclusion of multiple births is unlikely to significantly affect our assessment of intervention effects, and therefore we have chosen the analysis where the twin intra-class correlation coefficient will not be considered as the primary analysis.

**Discussion:**

In line with the Declaration of Helsinki and the International Conference on Harmonization Good Clinical Practice guidelines, we have developed and published this statistical analysis plan for the SafeBoosC III trial, prior to any data analysis.

**Trial registration:**

ClinicalTrials.org, NCT03770741. Registered on 10 December 2018.

## Background

Extremely preterm infants carry a high risk of death, with a mortality rate up to 25% [1, 2]. Furthermore, about 20% suffer from long-term neurodevelopmental impairment such as cerebral palsy or low intelligence quotient [2, 3]. Psychomotor impairment is a major cause of reduced quality of life and increased costs of medical care, rehabilitation, and special education in this population [4]. Low intelligence quotient affects all aspects of life. With increasing life expectancy, these combined prematurity-related factors pose a significant problem.

Hypoxia has been associated with mortality and brain injury in the preterm population [5]. In the SafeBoosC II trial, cerebral near-infrared spectroscopy (NIRS) monitoring combined with an evidence-based treatment guideline significantly reduced the burden of hypoxia during the first days of life in preterm infants [6]. There were also trends towards reduced occurrence of severe brain injury and mortality [6]. On the other hand, the incidence of bronchopulmonary dysplasia and retinopathy of prematurity was higher among NIRS-monitored neonates [6]. However, SafeBoosC II was not powered to demonstrate effects on these outcomes; thus, high-certainty evidence of clinical benefit and harm in extremely preterm infants is lacking [7]. We therefore plan a larger phase III trial, SafeBoosC III, powered to demonstrate the potential benefits and harms of treatment based on cerebral NIRS monitoring compared with treatment as usual on patient-centred clinical outcomes. As the SafeBoosC III trial will be conducted in compliance with the Declaration of Helsinki in its latest form and the International Conference on Harmonization Good Clinical Practice guidelines [8], we have developed this detailed statistical analysis plan. We believe this will decrease the risk of outcome reporting bias and data-driven analyses.

## Methods/design

### Trial overview

**Fig. 1:**
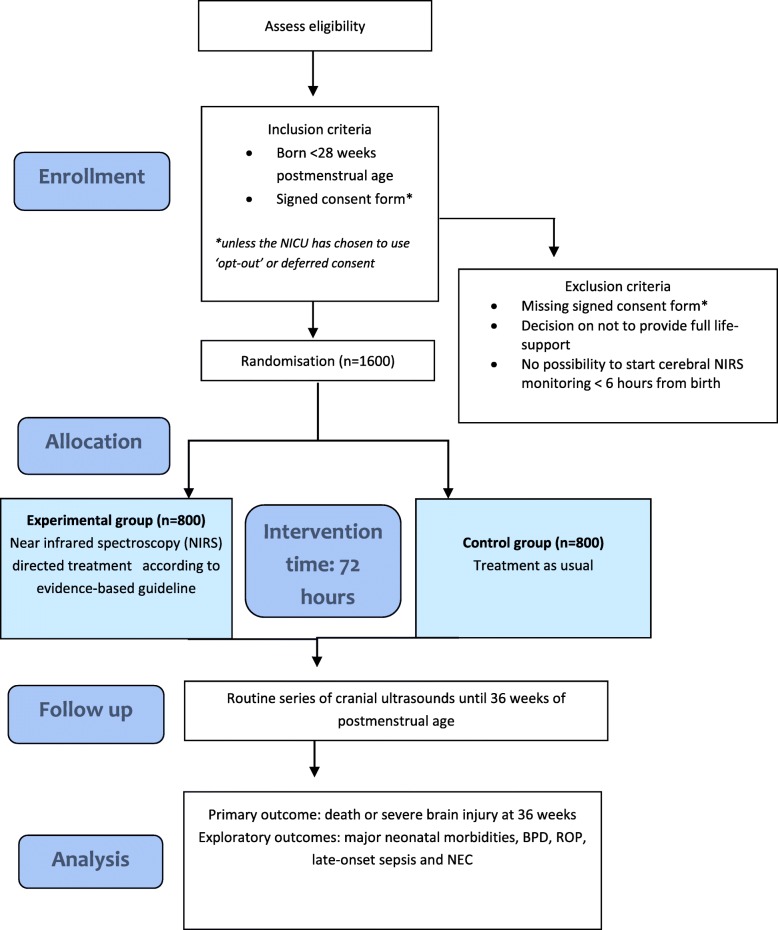
Trial flow diagram. BPD bronchopulmonary dysplasia, NEC necrotising enterocolitis, NICU neonatal intensive care unit, ROP retinopathy of prematurity

SafeBoosC III is an investigator-initiated, open-label, randomised, multinational, pragmatic phase III clinical trial with a parallel group design. The primary objective is to evaluate the benefits and harms of treatment based on cerebral NIRS monitoring during the first 72 postnatal hours in extremely preterm infants [9], compared with treatment and monitoring as usual, to reduce cerebral hypoxia [10]. The hypothesis is that treatment based on NIRS monitoring for extremely preterm infants during the first 72 h of life will result in a reduction in death or severe brain injury assessed at 36 weeks postmenstrual age. We plan to test for superiority of the experimental intervention compared with the control group for only the primary outcome, since exploratory outcomes will only be hypothesis generating (see ‘Level of significance’). Infants will be randomised with an allocation ratio of 1:1 to either the experimental group or the control group stratified for neonatal intensive care unit (NICU) and gestational age (lower gestational age (< 26 weeks) compared to higher gestational age (≥ 26 weeks)). Details of the randomisation method are held securely in the statistics master file. Infants in the experimental group will start cerebral NIRS monitoring as close to birth as possible, but at least within 6 h of birth, and receive treatment based on NIRS monitoring during the first 72 h of life (Fig. 1). These treatments will follow an evidence-based treatment guideline [11]. Infants in the control group will not receive cerebral NIRS monitoring and will be monitored and treated according to local guidelines and practices (i.e. treatment as usual). Due to the nature of NIRS, it is difficult to blind the clinical staff or the parents of the trial participants.

Three different consent methods may be used in this trial: prior informed consent (prenatal and postnatal); deferred consent; and prior assent/‘opt-out’. The trial will be conducted at more than 50 centres across up to 20 countries (16 European countries, India, China, and the USA), and the protocol will be published in an international peer-reviewed journal [10].

The SafeBoosC III trial is registered at ClinicalTrials.org (NCT03770741) and is compliant with the Declaration of Helsinki in its latest form and with the International Conference on Harmonization Good Clinical Practice. The trial will be approved by relevant authorities, including research ethics boards and data protection agencies, in all participating centres. The progression of the trial can be followed at www.safeboosc.eu. This statistical analysis plan has been written and submitted before randomisation commences and all data analysis for the main publication will be compliant to this plan.

### Outcomes

#### Primary outcome

The primary outcome is a composite of either death or severe brain injury. Severe brain injuries will be defined as grade III or IV cerebral haemorrhage (Papile’s classification) [12], cystic periventricular leukomalacia [2], cerebellar haemorrhage, post-haemorrhagic ventricular dilatation, or cerebral atrophy. These cerebral outcomes will be reported as detected on any one of a series of cranial ultrasound scans that are routinely performed in these infants.

Outcome assessment of mortality will not be blinded, but diagnosis and classification of brain injury and entry of this information into electronic case report forms will be conducted by a clinician blinded to group allocation.

#### Exploratory outcomes

- A count of the presence of the three major neonatal morbidities associated with neurodevelopmental impairment later in life [13]: bronchopulmonary dysplasia (defined below), retinopathy of prematurity (as defined below), and severe brain injury as defined in the primary outcome (i.e. a value of 0, 1, 2, or 3)
- Bronchopulmonary dysplasia defined as oxygen or ventilator/continuous positive airway pressure requirement at 36 weeks’ postmenstrual age
- Retinopathy of prematurity stage 3 and above at any time prior to 36 weeks’ postmenstrual age
- Late-onset sepsis (> 72 h after birth) defined as treatment with antibiotics for at least 5 days
- Necrotising enterocolitis stage 2 or higher using the modified Bell’s staging system [14] and/or focal intestinal perforation at any time up until 36 weeks’ postmenstrual age

#### Outcome assessment time point

All outcomes will be assessed at 36 weeks postmenstrual age.

### Sample size

We have calculated our sample size with an α of 5%, a power of 90%, and a ratio of experimental trial participants to control trial participants of 1:1. The primary outcome is the composite outcome of death or severe brain injury. Sample size calculations were performed for the composite outcome and not for the individual components.

Calculated from the 2009 dataset from the EuroNeoNet project [15] the mortality was 33% and severe intracranial haemorrhage was observed in 15%. In the SafeBoosC II trial, the proportion of trial participants in the control group with the same composite primary outcome was approximately 34% and in the experimental group was 26% [6]. Mortality was 24% in the control group versus 13% in the experimental group and the proportion of infants with severe brain injury was 23% versus 13% [6].

Based on the aforementioned, a total of 1600 infants — 800 infants randomised to the experimental group and 800 infants to the control group — would be required to demonstrate a reduction of the primary outcome from 34.0% to 26.5%, with an α of 5% and a power of 90%. This corresponds to a 22% relative risk reduction or a 7.5% absolute risk reduction. We consider this a clinically relevant and important benefit, since mortality is of direct patient relevance and since surviving infants with severe brain injury (about 25%) are at approximately 40% risk of moderate-to-severe neurodevelopmental impairment [16]. This absolute risk reduction corresponds to a ‘number-needed to treat’ of 15 infants and, if our null hypothesis is rejected, is likely to influence clinical practice.

### Power calculations for exploratory outcomes

For the exploratory outcomes, we have performed power calculations as presented in Table 1.

**Table 1:** Overview of power calculations for exploratory outcomes

Outcome	Assumption on prevalence in background population (%)	Assumption on risk increase or decrease (%)	Power (%)
Major neonatal morbidities	0.62 (0.8)^a^	20	87
Bronchopulmonary dysplasia	40	20	89
Retinopathy of prematurity	13	30	68
Late-onset sepsis	40	20	91.2
Necrotising enterocolitis	11	17	23

For definition of outcomes, see ‘Outcomes’. All power calculations have been made with a 5% significance level

^a^Presented as mean count (standard deviation)

Assuming a mean major neonatal morbidity count (bronchopulmonary dysplasia, retinopathy of prematurity, and severe brain injury) of 0.62 among extremely preterm infants [17], with a standard deviation of 0.80 and a relative risk increase or decrease of 20% in the experimental group, we will be able to detect this difference between the experimental and control group with 87% power at a 5% significance level (Table 1).

Assuming a 40% prevalence of bronchopulmonary dysplasia among extremely preterm infants [18] and a relative risk decrease or increase of 20% in the experimental group, we will be able to detect this difference between the experimental and control group with 89% power at a 5% significance level (Table 1).

Assuming a 13% prevalence of stage 3 and above retinopathy of prematurity among extremely preterm infants and a relative risk decrease or increase of 30% in the experimental group [7], we will be able to detect this difference between the experimental and control groups with 68% power at a 5% significance level (Table 1).

Assuming a 40% prevalence of late-onset sepsis in the control group [1], defined as treatment with antibiotics for at least 5 days, and a 20% relative risk decrease or increase in the experimental group, we will be able to detect this difference between the experimental and control groups with 91.2% power at a 5% significance level (Table 1).

Assuming an 11% prevalence of stage 2 and 3 necrotising enterocolitis among extremely preterm infants and a 17% relative risk decrease or increase in the experimental group, as is the estimate from existing trials [7], we will be able to detect this difference between the experimental and control groups with 23% power at a 5% significance level (Table 1).

### Assessment of outcomes and additional clinical variables

There will be three time points for data collection: at randomisation (from 0 to 6 h after birth); at the end of the intervention period (72 h of life); and at 36 weeks postmenstrual age. Data on feasibility will be assessed at randomisation. At the end of the intervention period, data collection will primarily reflect cerebral NIRS monitoring and safety parameters. As mentioned, all outcomes will be assessed at 36 weeks postmenstrual age. Severe brain injury diagnosis and classification data will be collected either by neonatologists assessing all cranial ultrasound scans performed up until 36 weeks postmenstrual age or by reading radiologists’ descriptions of these scans. This assessment and data entry will be conducted by a person blinded to group allocation. No long-term follow-up has been formally planned. However, we encourage clinical sites to conduct long-term follow-up, and we have therefore developed an appendix in the protocol (see full protocol at www.safeboosc.eu) describing possible outcomes for later follow-up studies and how these could be conducted. Currently, no protocol for such an ancillary study has been developed.

### Explanatory variables

Additional clinical data on trial participants will be drawn from clinical files, in order to compare characteristics between intervention groups. Data will be drawn from clinical records at 72 h of age and 36 weeks postmenstrual age. These data consist of a subset of explanatory variables, with the majority usually being reported to the neonatal network databases, such as Vermont Oxford Network [19]. These data will be presented in a table in the main publication (see Table 2). Tests of statistical significance will not be undertaken for explanatory variables. Categorical data will be summarised by numbers and percentages. Continuous data will be summarised by mean and standard deviation if normally distributed or by median and interquartile range if non-normally distributed.

**Table 2:** Explanatory variables divided by experimental group and control group participants

Variables	Experimental group (*n*)	Control group (*n*)
At randomisation		
Birth weight (g)		
Gestational age (weeks)		
Apgar 1 min (1–10)		
Apgar 5 min (1–10)		
Gender		
Male (%)		
Female (%)		
At 72 h of age		
Age when NIRS monitoring started (h)^a^		N/A
Stopping NIRS monitoring before end of monitoring period (%)^a^		N/A
Parents discontinuing trial participation (%)		
Changes in treatment due to cerebral hypoxia (%)^a^		N/A
Registered cardiovascular support treatment (%)^a^		N/A
Type of NIRS device used^a^ INVOS (%) NIRO (%) Fore-Sight (%) Sensmart (%) O3 (%) Egos (%) Oxyprem (%) Other (%)		N/A
Cerebral NIRS monitoring despite being in control group (%)^b^	N/A	
Surfactant therapy (%)		
Severe adverse reactions (%)		
At 36 weeks postmenstrual age		
Major congenital anomaly (%)		
Mechanical ventilation (%)		
Time with mechanical ventilation (days)		
Patent ductus arteriosus (%)		
Weight (g)		
Early cranial ultrasound scan (%)		
Late cranial ultrasound scan (%)		

Data expressed as median (range) for continuous variables, and numbers (percentage) for dichotomous variables

*N/A* not applicable, *NIRS* near-infrared spectroscopy

^a^Variables only relevant for experimental group participants

^b^Variables only relevant for control group participants

### Safety

We will report the total number of serious adverse reactions, as defined in the protocol [10] for each group, as well as the total number of participants who experienced one or more serious adverse reactions in each group. We will also report the total number of serious adverse events, as defined in the protocol [10] in each group, as well as the number of participants who experienced one or more serious adverse events in each group.

### Level of significance

The thresholds for significance will be assessed according to a 5-point procedure, suggested by Jakobsen et al. [20]. We will calculate and report confidence intervals and exact *p*-values for the primary and exploratory outcomes. All confidence intervals presented will be 95% and two-sided. A *p*-value of less than 0.05 will be used as the threshold for statistical significance for our primary outcome, since this value was used as the acceptable risk of type I error in our sample size estimation (see ‘Sample size’) and since we plan to report on only one primary outcome. However, in our interpretation of the results, we will assess any effect of the experimental intervention according to the point estimate taking into consideration the confidence interval as well as intervention effects on other outcomes [21]. All remaining outcome results will only be considered hypothesis-generating. Since our primary conclusion will be based on one outcome result at one time point, we will limit problems associated with multiple testing, due to multiple outcome comparisons [22].

Secondly, we will calculate and report the Bayes factor [23] for the primary outcome [24]. The Bayes factor is the ratio between the probability of the results given that the null hypothesis (H_0_) is true divided by the probability of the results given that the alternative hypothesis (H_A_) is true [23]. In the SafeBoosC III trial, the alternative hypothesis is that the treatment effect is the effect that was used for the sample size calculation: a 22% relative risk reduction in the experimental group. By calculating the Bayes factor, we will be able to interpret the results of the primary outcome in relation to former trial results [6].

Thirdly, Lan–DeMets monitoring boundaries will be used to adjust the threshold for statistical significance at each interim analysis to judge whether the trial should be terminated early [25]. This is done in order to avoid a false rejection of the null hypothesis based on insufficient sample sizes [26]. The trial will not be stopped prematurely due to futility. The fourth step in the five-step procedure by Jakobsen et al., regarding adjustment of *p*-values based on multiple testing of the primary outcome, is not applicable to our trial, since we have a single primary outcome [20].

We will take the upper and lower limits of the confidence intervals into consideration when making study conclusions [21]. Clinical significance will be assessed by calculating the number needed to treat based on the absolute risk reduction data. Based on the results from the phase II trial, we expect an absolute risk reduction of 7.5%, which corresponds to a number needed to treat of 15 (see ‘Sample size’) [6].

### Interim analyses

One pre-planned interim analysis will be conducted after one-third of trial participants have been randomised. The timing and prevalence of additional interim analyses will be decided solely by the data monitoring and safety committee members. The data monitoring and safety committee will make recommendations to the steering group to continue, change, hold, or terminate the trial. This recommendation will be based primarily on safety considerations and will be guided by statistical monitoring guidelines, defined in the data monitoring and safety committee charter. The data monitoring and safety committee will be provided with the following data from the Coordinating Data Centre: number of participants randomised, number of participants per intervention group (0,1), number of participants stratified per stratification variable per intervention group (0,1), and number of events (primary outcome, SAEs, and SARs) in the two groups. Based on the evaluations of these outcomes, the data monitoring and safety committee will decide whether they want further data from the Coordinating Data Centre, and when next to perform analyses of data. Based on the analyses of the safety variables, the data monitoring and safety committee is suggested to use Lan–DeMets sequential monitoring boundaries, based upon a relative risk increase of 100% of mortality from 25% to 50%. For any of the other safety outcomes, the statistical limit to guide its recommendations regarding early termination of the trial for harms is recommended also to be conservative.

### Handling of missing data

Missing data will be minimised by performing repeated monitoring of data entry into electronic case report forms. In this way, we will be able to monitor the extent of missing data and intervene if necessary. Hence, we do not anticipate that there will be any significant number of missing values. However, we will consider using multiple imputation and present best–worst and worst–best case scenarios if it is not valid to ignore missing data according to the standards reported by Jakobsen et al. [27]. When using best–worst and worst–best case scenarios, we will assess the potential range of impact of the missing data for the trial results [27]. In the ‘best–worst’ case scenario, it is assumed that all patients lost to follow-up in the experimental group have had a beneficial outcome, and all those with missing outcomes in the control group have had a harmful outcome [27]. Conversely, in the ‘worst–best’ case scenario, it is assumed that all patients who were lost to follow-up in the experimental group have had a harmful outcome, and that all those lost to follow-up in the control group have had a beneficial outcome [27].

As recommended, we will describe reasons why outcome data are missing in the main study manuscript [28]. Furthermore, we will compare explanatory variables between all participants randomised to intervention groups (including those with missing outcomes), and also between participants in the intervention groups, where outcomes are reported. This is done to identify imbalances between groups due to missing outcome data [29].

### Twins and their intra-cluster correlation

In extremely preterm populations, 30% of births may be twins [6], which poses a potential problem for statistical analyses as the outcomes among pairs of twins are potentially correlated [30]. In the SafeBoosC III trial, multiple birth infants will be randomised as a ‘pair’ or a ‘group’ (i.e. all siblings will be allocated to the same intervention group). In centres where only one or two cerebral monitoring devices are available, it may not be possible to include all infants from multiple births. Thus, only one of a pair or only one or two infants of triplets may be included. The sibling(s) enrolled in the trial will be the one(s) born last. In the SafeBoosC II trial, the intra-class correlation coefficient (ICC) of the burden of hypoxia within pairs of twins was negligible. The ICC for various binary outcomes has been estimated in a previous study: ICC for death before discharge was estimated as 0.00 (95% confidence interval (CI) –0.04 to 0.02) and for intraventricular haemorrhage grade 3 or 4 as − 0.01 (95% CI − 0.05 to 0.01) [31]. These values correlate to a design effect very close to 1 [31]. Therefore, in the primary analysis, we will analyse twin data as independent observations. However, due to the possibility that the correlation between the primary outcome within multiple births will interfere with the estimation of the treatment effect [32], and particularly the assessment of estimation uncertainty, we will perform a sensitivity analysis, taking this effect into consideration. This sensitivity analysis will be performed using the generalised estimating equation (GEE) approach utilising an exchangeable covariance matrix with site (NICU) and stratification variables as fixed effects. The results of both primary outcome analyses will be presented and discrepancies between the two analyses discussed in the final publication. Furthermore, we will calculate, report, and discuss the ICC for the primary outcome.

### Stratification

We will use site (NICU) and gestational age (lower gestational age (< 26 weeks) compared to higher gestational age (≥ 26 weeks)) as stratification variables in the randomisation. Analyses for all outcomes will be adjusted for these stratification variables [33–35].

### Assessment of underlying statistical assumptions

For all regression analyses, we will test for major interactions between each covariate and the intervention variable. We will, in turn, include each possible first-order interaction between included covariates and the intervention variable. For each combination, we will test whether the interaction term is significant and assess the effect size. We will only consider that there is evidence of an interaction if the interaction is statistically significant after Bonferroni-adjusted thresholds (0.05 divided by number of possible interactions) and if the interaction shows a clinically significant effect. If it is concluded that the interaction is significant, we will be presenting an analysis separately for each (e.g. for each site if there is significant interaction between the trial intervention and ‘site’) and an overall analysis including the interaction term in the model.

### Assessment of underlying statistical assumptions for dichotomous outcomes

We will assess whether the deviance divided by the degrees of freedom is significantly larger than 1 to assess for relevant overdispersion, and in this case consider using a maximum likelihood estimate of the dispersion parameter. To avoid analytical problems with either zero events or problems such as all participants dying at a given site, we have only included sites planning to randomise a sufficient number of participants. However, we cannot exclude the risk that some sites might have problems with recruitment. We will, by checking whether the number of participants is larger than 10 (rule of thumb) per site, pool the data from small sites if the number of participants is too low.

### Statistical analyses

Analyses will be made on the intention-to-treat population for all outcomes, since this method maintains baseline comparability of the intervention groups [29]. The intention-to-treat population will include all randomised patients, regardless of missing data, lost to follow-up or adherence to the intervention.

In our primary analysis, we will analyse dichotomous outcomes using mixed-effect logistic regression and count data using mixed-effect linear regression with robust standard errors. In all regression models, ‘site’ will be included as a random effect. The remaining stratification variables (age and intervention groups) will be included as fixed effects. The sensitivity analysis accounting for the possible correlation between twins is described in ‘Twins and their intra-cluster correlation’.

As an additional sensitivity analysis, we will perform a per-protocol analysis, only including participants who had no missing data, were not lost to follow-up, and adhered to the intervention. Adherence to the intervention is defined as continuous cerebral oxygenation monitoring during the first 72 h of life or until death.

We will, in a secondary analysis, analyse the results using random-effects meta-analysis [36].

All outcomes will be analysed collectively since the follow-up time is identical.

### Data management

The data management plan has been described in the protocol paper [10].

### CONSORT flow diagram

The main publication will include a Consolidated Standards of Reporting of Randomised Trials (CONSORT) flow diagram, following the CONSORT 2010 Statement [37]. This will be used to summarise the number of patients who were randomised, allocated to the experimental and control groups, adhered and unadhered to the intervention, lost to follow-up (including parental and physician withdrawal), randomised and included in the primary analysis, and randomised and excluded from the primary analysis.

### Withdrawal

Parents will be able to withdraw consent at any time during the trial. However, data on participants up until the day of withdrawal will be used and participants will be part of the intention-to-treat population and analysis.

### Blinding of statisticians

All data managers, statisticians, and those drawing conclusions will be blinded to treatment allocation. Two blinded statisticians connected to The Copenhagen Trial Unit will independently perform all statistical analyses and the two statistical reports will be published as supplemental material. Discrepancies between the two reports will be discussed by the Steering Committee of the trial. The two intervention groups will be coded ‘A’ and ‘B’. When comparability between the two independent analyses have been obtained, two abstracts will be written: one assuming ‘A’ is the experimental group and ‘B’ is the control group – and one assuming the opposite. After the conclusions have been drawn, blinding will be broken, and the final manuscript will be based on the correct pre-written abstract.

### Simulation of twin scenarios

To explore the potential impact of twin correlation, we conducted a simulation study to assess potential impact on power and coverage probabilities of confidence intervals (i.e. does the computed 95% CI contain the true parameter values with 95% probability). We compared the naive analysis (primary analysis of the primary outcome), which ignores twin pairs, to a GEE-based approach which does account for twin correlation. We did this by simulating 10,000 trials with sample size and true parameter values as in the sample size estimation and varied twin probability and ICC. These results are presented in Table 3. This simulation study shows that for a low ICC value or low twin proportion, we can expect both the naive and GEE analyses to have correct coverage and equal power. For a high ICC and a high twin proportion, we can expect the GEE analysis to retain correct coverage, while the naive analysis will have decreased coverage; these differences, however, would be minimal. For high twin proportion and high ICC values, the effective sample size was reduced, which as expected implied that the correct analysis (the GEE) yields a lower power than the intended 90%, albeit only marginally so, and that the coverage for the naïve analysis was a bit too low.

**Table 3:** Simulation study to assess power and coverage probabilities of confidence intervals of primary outcome

ICC	Proportion of twins	Power of naive analysis	Power of GEE analysis	Coverage probability of naive analysis	Coverage probability of GEE analysis
0	0.1	0.91	0.91	0.95	0.95
0	0.2	0.91	0.91	0.95	0.95
0	0.3	0.90	0.90	0.95	0.95
0	0.4	0.90	0.90	0.95	0.95
0.01	0.1	0.91	0.91	0.95	0.95
0.01	0.2	0.90	0.90	0.95	0.95
0.01	0.3	0.90	0.90	0.95	0.95
0.01	0.4	0.91	0.90	0.95	0.95
0.03	0.1	0.90	0.90	0.95	0.95
0.03	0.2	0.91	0.91	0.95	0.95
0.03	0.3	0.90	0.90	0.95	0.95
0.03	0.4	0.90	0.90	0.95	0.95
0.13	0.1	0.90	0.90	0.95	0.95
0.13	0.2	0.90	0.89	0.94	0.95
0.13	0.3	0.90	0.89	0.94	0.95
0.13	0.4	0.90	0.88	0.94	0.95
0.2	0.1	0.90	0.90	0.95	0.95
0.2	0.2	0.90	0.89	0.94	0.95
0.2	0.3	0.90	0.88	0.94	0.95
0.2	0.4	0.89	0.87	0.94	0.95

*GEE* generalised estimating equation, *ICC* intra-class coefficient

## Discussion

This article presents the detailed statistical analysis plan for the SafeBoosC phase III trial. It has been developed and submitted prior to any randomisation or data collection in order to avoid data-driven analyses and outcome reporting bias. Data will be analysed on the intention-to-treat population, and multiple imputations will be used if the proportion of missing data cannot be ignored (see ‘Handling of missing data’). An anonymised dataset regarding all outcomes will be uploaded to a public database to be available for other researchers and peers 6 months after acceptance of the study manuscript.

We plan to report on both primary and exploratory outcomes in the main publication, but the conclusion will solely be based on the results of the primary outcome. If the result is statistically insignificant, based on the 5-point procedure by Jakobsen et al. [20], we will conclude that there is no significant difference between the intervention and treatment as usual (see ‘Level of significance’).

### Dealing with multiple analyses

Planning multiple analyses on a primary outcome has the potential to increase the risk of type I errors, due to multiple testing [38]. If it is predefined that a significant difference between the experimental and control groups on any one of the primary outcome analyses is sufficient to declare superiority of a given intervention, one would have to correct for multiple testing by decreasing the α value [22, 39]. On the other hand, planning that all primary outcome analyses must show significant benefit of the intervention to declare superiority has the potential to increase the risk of type II errors, due to insufficiently powered analyses [40]. Hence, by only planning one analysis for the primary outcome and by defining additional analyses as sensitivity analyses (see ‘Twins and their intra-cluster correlation’), we have eliminated the type I and type II error-related issues described above. The sensitivity analyses on the primary outcome will only be used to discuss and illustrate the results of the primary analysis.

### Strengths

According to our knowledge, SafeBoosC III will provide the largest trial, thus far, evaluating the benefits and harms of treatment guided by cerebral NIRS monitoring – not only in extremely preterm infants [41] but across all patient populations [42].

It is an important strength that both the protocol and statistical analysis plan have been developed and submitted prior to any randomisation or data collection [8, 10]. Furthermore, we have also taken the issue of twins and their intra-cluster correlation into consideration, by performing an additional sensitivity analysis to address its potential effect on results (see section on ‘Twins and their intra-cluster correlation’). To address the potential impact of twin correlation on our results, we also performed a simulation study, showing that we can expect the potential impact of twin correlation to be minor.

There is genuine evidence that most randomised clinical trials lack external validity, which is an important explanation for why multiple interventions proven beneficial in randomised clinical trials are underused in routine clinical practice [43]. Since SafeBoosC III is an international trial including multiple sites across different countries, limitations to external validity such as different practices between countries and health-care systems seems less of an issue for external validity. Furthermore, the external validity of our results will also be described in the main publication, as recommended in the Consolidated Standards of Reporting of Randomised Trials guidelines [37].

### Limitations

Our methodology also has limitations. Only three of the five exploratory outcomes are sufficiently powered (80% power) to show a significant difference between the experimental and control groups, at a 5% significance level. If these were categorised as secondary or additional primary outcomes, we would need to correct for multiple testing by decreasing the α value using Bonferroni adjustments [39]. Therefore, we will not make any clinical conclusions based on these results. However, we believe they are important to report and assess since they represent major neonatal morbidities in our study population [1, 13, 44].

As thoroughly reported in the SafeBoosC III design paper [10], it is difficult to blind clinical staff, the infant, and the parents of the trial participants, which introduces risks of bias [45–48]. This important concern is discussed in detail in our design paper [10].

As recommended in the European Medicines Agency Guidelines on Multiplicity Issues in Clinical Trials, the components of the primary composite outcome (i.e. death and severe brain injury) will be analysed separately [49]. However, interpretation of these sub-analyses will be difficult, since death and severe brain injury as individual outcomes are insufficiently powered to show a real benefit of the intervention.

## Trial status

At present, the study protocol has been registered at www.clinicaltrials.gov (NCT 03770741, registered on 10 December 2018) and has been accepted for publication [10]. The first participant was randomised on 27 June 2019. Status on recruitment can be accessed at www.safeboosc.eu.

### Statistical analysis plan status

Version 1.0 (8 August 2019). This document has been written based on information available in the protocol paper [10].

## Data Availability

Not applicable.

## Abbreviations

CI: Confidence interval
GEE: Generalised estimating equation
ICC: Intra-class correlation coefficient
NICU: Neonatal intensive care unit
NIRS: Near-infrared spectroscopy
